# Facilitators and barriers for lifestyle change in people with prediabetes: a meta-synthesis of qualitative studies

**DOI:** 10.1186/s12889-022-12885-8

**Published:** 2022-03-21

**Authors:** Gyri Skoglund, Birgitta Blakstad Nilsson, Cecilie Fromholt Olsen, Astrid Bergland, Gunvor Hilde

**Affiliations:** 1 Department of Physiotherapy, Faculty of Health Sciences, OsloMet- Oslo Metropolitan University, Oslo, Norway; 2 Section for Physiotherapy, Division of Medicine, Oslo University Hospital, Oslo, Norway

**Keywords:** Prediabetes, Lifestyle change, Risk perception, Intrinsic motivation, Maintenance, Self-determination, Self-regulation, Ecological model

## Abstract

**Background:**

The increasing prevalence of type 2 diabetes worldwide is a major global public health concern. Prediabetes is a reversible condition and is seen as the critical phase for the prevention of type 2 diabetes. The aim of this study is to identify and synthesize current evidence on the perceived barriers and facilitators of lifestyle change among people with prediabetes in terms of both initial change and lifestyle change maintenance.

**Methods:**

A systematic literature search in six bibliographic databases was conducted in April 2021. Potential studies were assessed for eligibility based on pre-set criteria. Quality appraisal was done on the included studies, and the thematic synthesis approach was applied to synthesize and analyse the data from the included studies.

**Results:**

Twenty primary studies were included, containing the experiences of 552 individuals. Thirteen studies reported participants perceived facilitators and barriers of lifestyle change when taking part in community-based lifestyle intervention programs, while seven studies reported on perceived facilitators and barriers of lifestyle change through consultations with health care professionals (no intervention involved).

Three analytical themes illuminating perceived barriers and facilitators for lifestyle change were identified: 1) the individual’s evaluation of the importance of initiating lifestyle change*,* 2) the second theme was strategies and coping mechanisms for maintaining lifestyle changes and 3) the last theme was the significance of supportive relations and environments in initiating and maintaining lifestyle change*.*

**Conclusion:**

Awareness of prediabetes and the perception of its related risks affects the motivation for lifestyle change in people at risk of type 2 diabetes; but this does not necessarily lead to lifestyle changes. Facilitators and barriers of lifestyle change are found to be in a complex interplay within multiple ecological levels, including the interpersonal, intrapersonal, environmental and policy level. An integrated understanding and analysis of the perceived barriers and facilitators of lifestyle change might inform people with prediabetes, healthcare professionals, and policy makers in terms of the need for psychological, social, and environmental support for this population.

**Supplementary Information:**

The online version contains supplementary material available at 10.1186/s12889-022-12885-8.

## Background

Type 2 diabetes represents a significant global health burden, with great impact on individuals, families, and societies. The prevalence of type 2 diabetes is increasing worldwide. Reports estimate that 578 million people will have diabetes in 2030, and the number will increase by 51% (700 million) in 2045 without urgent and sufficient action [1]. Considering the growing epidemic of diabetes and its complications, the increasing prevalence of prediabetes is a major global public health concern [2]. The term prediabetes is used to identify those individuals who are at risk of future diabetes and it is also associated with an increased cardiometabolic risk [2]. Prediabetes is a condition characterized by elevated blood glucose levels, below the threshold limit for type 2 diabetes but above normal levels, and it is estimated that 70% of individuals with prediabetes will eventually develop diabetes [2, 3]. Prediabetes is seen as the critical phase for prevention, as the patients’ condition at this stage is reversible and could therefore serve as a window of opportunity to combat type 2 diabetes [3].

The risk of developing prediabetes increases with being overweight, living a sedentary lifestyle, age, and having a family history of diabetes [4]. Lifestyle changes aiming for healthy behaviour in terms of healthy diet, regular physical activity, and maintaining a healthy body weight are the cornerstones of prevention or the delayed onset of type 2 diabetes [4, 5]. Weight reduction is shown to be the single-most important factor in reducing diabetes incidence: for every kilogram of weight loss, diabetes incidence has been reduced by 16 percent [6]. Several studies have shown the efficacy of lifestyle intervention with regards to diabetes prevention, with a relative risk reduction of 36–54% in those with prediabetes [7]. The positive outcomes of lifestyle changes have been observed in diverse populations [7, 8], and diabetes prevention has therefore become a key priority for many nations, forming the basis of many national and international practice guidelines [9–11]. Although research has shown that lifestyle intervention programs are effective [7, 8, 12, 13], improvements over the long term have been shown to deteriorate, highlighting challenges with long-term adherence and the maintenance of lifestyle changes [5]. A systematic review of obesity-related lifestyle change interventions, has shown that health behaviours that are initiated and regulated via autonomous motivation are more likely to be maintained over time through autonomous motivation, self-efficacy, and self-regulation skills [14].

### Theoretical framework

In addition to previous research, the theoretical understanding of lifestyle and behavior change is important. A systematic review by Kwasnicka et al. [15] identified and synthesized 100 current theoretical explanations for behavioral change and maintenance. The review stated that there are distinct patterns of theoretical explanation for initial change and change maintenance and they highlighted the differential nature and role of five overarching, interconnected themes: maintenance motives, self-regulation, resources (psychological and physical), habits, and environmental and social influences. The individual’s motivation is crucial for behaviour change and maintenance, and motives that initiate change may differ from those maintaining change [15]. Approaches to initiate behaviour change can include motivation in the form of external pressure or control or the positive use of incentives or rewards, but these approaches are often insufficient in order to enhance maintenance of lifestyle change [16].

### The ecological model

In addition to the theoretical explanations of Kwasnicka et al. [15] the ecological model can be a helpful framework in understanding the facilitators and barriers of lifestyle change in people with prediabetes in a larger context, and within a comprehensive understanding of the multiple determinants of health behaviours [17]. Health behaviours are dynamic, varying over individual’s lifespans, across settings, and over time [18], and the complex interplay of facilitators and barriers for healthy behaviours make lifestyle changes challenging to perform [19, 20]. According to ecological models of health there are multiple levels that influence on health behaviour and these are the intrapersonal, interpersonal, environmental, and societal level [21] and the barriers and facilitators for healthy behaviours constantly interact across all these levels [17]. In addition to the individual motivation and skills for lifestyle change, the ecological perspective further addresses the environmental aspect in understanding the facilitators and barriers in play, and how they impact on lifestyle change and maintenance [21].

In a review of qualitative studies by Kelly et al. [22] on the facilitators and barriers for healthy behaviours in midlife (40–64 years), they found that examples of consistent barriers included entrenched attitudes and behaviours, a lack of knowledge, a lack of time, lack of access to transport to facilities and resources, restrictions in the physical environment, and financial costs. The facilitators of healthy behaviour included enjoyment, health benefits, social support, and clear messages. Among the included qualitative studies, however, there were none specifically addressing those with prediabetes.

Former research has found that people who were aware of their prediabetes status were more likely to report a perceived threat of developing diabetes, but they did not report increased engagement in health behaviours [23–25]. This indicates the need to better understand what characterizes the facilitators and barriers for lifestyle change and maintenance in people with prediabetes, and by identifying this, research on lifestyle change and the implementation of health interventions can be optimally tailored and effective.

### Aim of the meta-synthesis

To our knowledge, no previous meta-syntheses examining perceived barriers and facilitators of lifestyle change among people at risk of developing type 2 diabetes have been performed. Hence, the current study aimed to identify and synthesize current qualitative evidence on facilitators and barriers of initial lifestyle change and maintenance based on the experiences of people with prediabetes.

## Methods

Meta-synthesis, or qualitative evidence synthesis, is the synthesis of primary research studies that relate to a specific topic in order to arrive at a new or enhanced understanding of a specific phenomenon being explored [26]. One approach to the synthesis of the findings of qualitative research is thematic synthesis as described by Thomas and Harden [27]. This method combines approaches from both meta-ethnography and grounded theory and was originally developed to guide review of intervention needs, appropriateness, and effectiveness [26, 28]. The approach of thematic synthesis is based on the method of thematic analysis used in primary qualitative research, however thematic synthesis enables new insights, interpretations and theories to be developed that has not been seen in the primary studies [29]. This meta-synthesis was prospectively registered with the International Prospective Register of Systematic Reviews (PROSPERO) (ID: CRD42020180051). We followed the Enhancing Transparency of Reporting the Synthesis of Qualitative Research (ENTREQ) framework [30].

### Search strategy

Systematic comprehensive literature searches were conducted in six bibliographical databases: Medline, Embase PsychInfo, CINAHL, Web of Science, and Cochrane. This choice of databases is in line with suggestions presented in the systematic review on optimal database combinations for literature searches in systematic reviews [31]. The searches were done by the first author (GS) with close assistance from a health research librarian. The search strategy aimed to cover primary studies addressing the study population of interest, phenomena of interest, and setting of interest; we limited the search to qualitative studies (see Additional file 1). The literature search was initially developed in Medline and afterwards translated to the other databases’ search syntax with both text words and adapted thesaurus terms. We also screened the reference lists of the included studies and related systematic reviews to identify further papers. Non-English studies were excluded to prevent cultural and linguistic bias in translations, and there was no publication year limit. The review includes data for studies identified in searches up to April 21st, 2021.

### Selection criteria

The primary studies were selected according to the study population, phenomenon of interest, setting and study design. An explicit description of criteria for inclusion and exclusion is presented in Table 1. The phenomenon of interest of this meta-synthesis was facilitators and barriers to lifestyle change and maintenance in people with prediabetes. When selecting the primary studies, we presumed that the facilitators and barriers could be identified from the data in the studies, but it did not necessarily have to be explicitly mentioned. The primary studies included according to the setting criteria, involved several studies where experiences from participation in a structured lifestyle intervention program were reported. The lifestyle interventions described in these studies mainly focused on physical activity and dietary change and weight loss.

**Table 1:** Eligibility criteria

**Criteria**	**Inclusion**	**Exclusion**	**Search Element ͣ**
Population	• People who recently (within one year) have been screened for risk of developing type 2 diabetes and diagnosed with prediabetes detected by measuring HbA1c level or fasting plasma glucose, or with an oral glucose tolerance test [32] Note: If Studies had a mixed population of both type 2 diabetes and prediabetes, and their findings on participants with prediabetes and type 2 diabetes could be read separately. We would include their study data on prediabetes participants • People aged > 18 years and over living in a home-based environment	• People diagnosed with type 2 diabetes • Women with gestational diabetes	Prediabetic State Prediabetes Impaired fasting glucose Hyperglycaemia Glucose intolerance Insulin resistance
Phenomenon of interest	With respect to the individual, interpersonal, and societal level: • Facilitators and barriers of initial lifestyle change • Facilitators and barriers of lifestyle change maintenance		Health behaviour change Lifestyle change
Setting	• The informants live in home-based environments and receive or have received support from health care providers within the community health care setting regarding lifestyle change • The informants may or may not have participated in a structured community-based lifestyle intervention program	• Studies reporting from hospital or institutional settings exclusively	Lifestyle intervention program Health behaviour intervention program
Study design	• Studies with qualitative analysis based on data from interviewing people at risk of developing type 2 diabetes • Mixed methods studies where the qualitative results are clearly separated from the quantitative data	• Qualitative studies where no human subjects participated and studies with primarily observational methods • Studies not published in peer reviewed journals	Qualitative studies
Time frame	• No set time frame		
Language	• Studies written in English and Scandinavian	• All other languages	

ͣAll Mesh terms and text words are listed in the search string in Additional file 1

One researcher (GS) screened all titles and abstracts retrieved from the literature search results, excluding studies that did not meet the inclusion criteria. The full texts of potentially relevant articles were then screened independently by two authors in groups of pairs (GS and AB, GS and GH, GS and BBN), and additional information was sought from the authors of the full text articles where necessary. If consensus was not reached between the two researchers, a third reviewer was consulted.

### Quality appraisal

Two authors in groups of pairs (GS and AB, GS and GH, GS and BBN) conducted a quality assessment of the included studies independently according to the Critical Appraisal Skills Program (CASP) checklist for qualitative research [33]. The checklist of ten questions allowed for the systematic appraisal of the qualitative research evidence included in our review (Table 2). The checklist guides the reviewer when assessing the validity, result and relevance of each study. After this initial independent assessment, the results of the appraisal were discussed, and a third reviewer was consulted to resolve any disagreements. There was an agreement that no studies were to be excluded based on the quality appraisal. However, an assessment of methodological quality would provide transparency and understanding of the relative strength and weaknesses of the body of evidence included [29].

**Table 2:** CASP: Quality appraisal results of the included primary studies

**Author, Country**	Was there a clear statement of the research?	Is a qualitative methodology appropriate?	Was the research design appropriate to address the aims of the research?	Was the recruitment strategy appropriate for the aims of the research?	Was the data collected in a way that addressed the research issue?	Has the relationship between the researcher and participants been adequately considered?	Have ethical issues been taken into consideration?	Was the data analysis sufficiently rigorous?	Is there a clear statement of the findings?	How valuable is the research/ will the results help locally?	
Abel et al., 2018, New Zealand [34]	Y	Y	Y	Y	Y	Y	Y	Y	Y	Y	10/10
Andersson et al., 2008, Sweden [35]	Y	Y	Y	Y	Y	Y	Y	Y	Y	Y	10/10
Coppell et al., 2017, New Zealand [36]	Y	Y	Y	Y	U	Y	U	U	Y	Y	7/10
Dyer et al., 2020, USA [37]	Y	Y	Y	Y	Y	N	N	Y	Y	Y	8/10
Hansen et al., 2010, Norway [38]	Y	V	Y	U	Y	N	Y	Y	Y	Y	8/10
Jallinoja et al., 2008, Finland [39]	Y	Y	Y	Y	Y	N	Y	Y	Y	Y	9/10
Korkiangas et al., 2011, Finland [40]	Y	Y	Y	Y	Y	Y	Y	Y	Y	Y	10/10
Kullgren et al., USA, 2015 [41]	Y	Y	Y	Y	Y	U	Y	Y	Y	Y	9/10
Kuo et al., 2013, Taiwan [42]	Y	Y	Y	Y	Y	Y	Y	Y	Y	Y	10/10
Lim et al., 2020, Singapore [43]	Y	Y	Y	Y	Y	Y	Y	Y	Y	Y	10/10
Lim et al., 2019, Singapore [44]	Y	Y	Y	Y	Y	Y	Y	Y	Y	Y	10/10
Mayega et al., 2014, Uganda/ Sweden [45]	Y	Y	Y	Y	Y	U	Y	Y	Y	Y	9/10
Morrison et al., 2014, Scotland [46]	Y	Y	Y	U	Y	Y	Y	Y	Y	Y	9/10
Penn et al., 2008, England [47]	Y	Y	Y	Y	Y	U	Y	Y	Y	Y	9/10
Strachan et al., 2018, Canada [48]	Y	Y	Y	Y	Y	Y	Y	Y	Y	Y	10/10
Bean et al.,2020 Canada [49]	Y	Y	Y	Y	Y	Y	Y	Y	Y	Y	10/10
Katangwe et al., 2020, England [50]	Y	Y	Y	Y	Y	Y	Y	Y	Y	Y	10/10
Griauzde et al., 2020, USA [51]	Y	Y	Y	Y	Y	N	Y	N	U	Y	7/10
Howells et al., 2021, England [52]	Y	Y	Y	Y	Y	U	Y	Y	Y	Y	9/10
Wallace et al., 2021, USA [53]	Y	Y	Y	Y	Y	N	Y	U	Y	Y	8/10

*Y* Yes, *U* Unsure, *N* No

### Data extraction and synthesis

The data extracted from the primary studies included all the text in the studies’ results chapters, including participant quotations. The extracted text was entered verbatim into NVivo Pro 12 (NVivo qualitative data analysis software; Melbourne, Australia: QSR International Pty Ltd., 2018). Each study was read several times to ensure that all the extracted text was related to the perspectives and experiences of people with prediabetes.

We used the thematic synthesis approach by Thomas and Harden [27], and this involved three main stages:

- 1) Line-by-line coding of the findings of the primary studies:
  Two independent reviewers performed an inductive line-by-line coding of the extracted material. New codes were generated independently of the original codes used in the primary studies. The codes were compared, and all codes that represented similarities across the primary studies and belonged to the same concept were organized into categories.
- 2) Development of descriptive themes:
  Descriptive subthemes were formed through the merging and grouping of categories in an iterative process, staying close to the primary data in the included studies. The primary studies were read and reviewed by GS to ensure that the descriptive themes captured and reflected the depth of the data reported in the primary studies.
- 3) Development of analytical themes:
  The descriptive themes were discussed in the research team in relation to the research question and organized within the main analytical themes. This was an iterative and cyclic process. In the analytical stage of the synthesis, we wanted to go beyond the descriptive findings trying to generate new understanding. After the development of the analytical themes, we related this to a higher-level theoretical framework to illuminate the central themes in the synthesis.

### Meta-synthesis researchers’ background and preconceptions

The research team consisted of two PhD students (GS and CFO) and three researchers with a clinical and academic background, all of whom were physiotherapists (AB, GH, and BBN). Although the authors acknowledge that there has been much debate regarding the definition of prediabetes and share some of the expressed concerns in the literature regarding the usefulness of this label [54, 55], the present analysis did not assume a critical stance toward this diagnosis, as our main aim was to use it as a descriptive category that would allow us to identify and review the existing literature in this area and on this population. It was the first author’s preunderstanding that risk perception is crucial in the initiation of lifestyle changes and that prediabetes might be a particularly challenging state in this respect. Furthermore, the researchers shared the preunderstanding that lifestyle change is complex and cannot be completely understood within a biomedical perspective. We used reflexive discussions to become aware of these preconceptions and reduce their influence on the analysis. However, in line with the qualitative research paradigm [56], we also acknowledge that they inevitably influenced the synthesis.

## Results

### Literature search results

**Fig.1:**
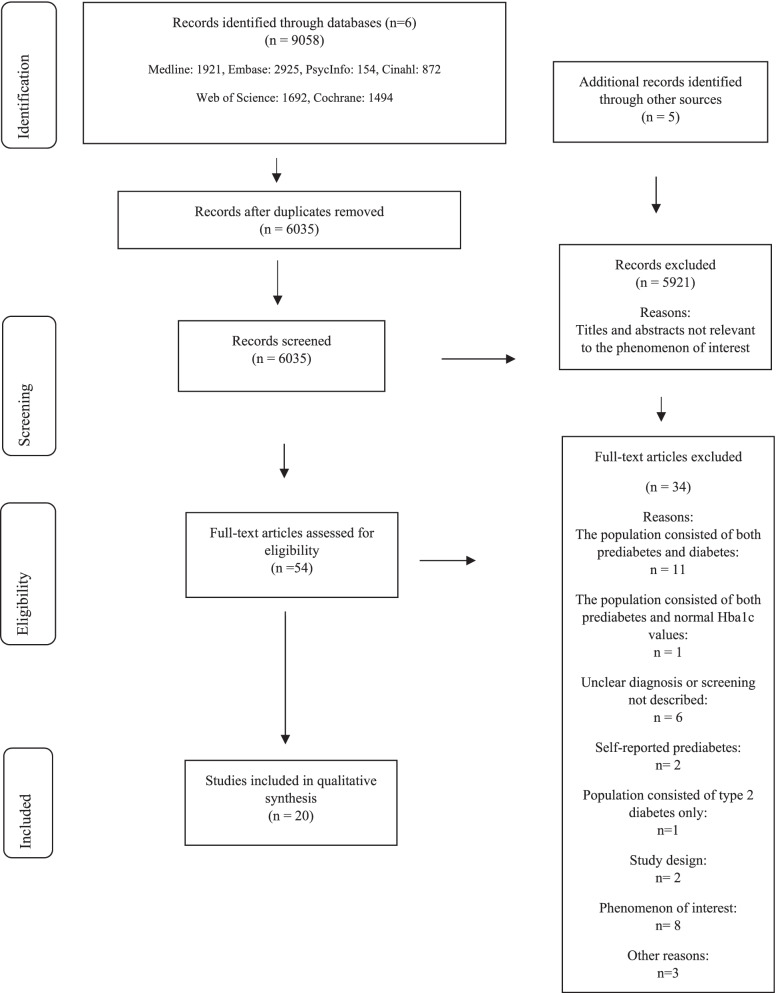
PRISMA Flow Diagram-identification and selection of studies [57]

The literature search resulted in 9058 identified studies and, after duplicates were removed, 6035 studies. Titles and abstracts were screened by the first author (GS), and, of these, 54 full-text articles were found to be considered eligible. These were screened by two independent reviewers according to pre-set criteria for inclusion and exclusion, and 20 studies were finally included; see PRISMA flow diagram (Fig. 1).

### Study characteristics

The 20 included studies were published between 2008 and 2021 and involved 552 participants in total. The age of the participants ranged from 21–79 years; 312 participants were women and 240 were men. All participants had been diagnosed with prediabetes within the last year (when the data was collected). Eight studies were from Europe, three from Asia, two from the South Pacific, four from the USA, two from Canada, and one from Africa. Each study was systematically assessed for its research question or statement of purpose, research method, theoretical framework, sample size, and setting. The characteristics of the 20 studies included in the thematic synthesis are presented in Table 3.

**Table 3:** Characteristics of the 20 included studies

Author, Country	Aim of study	Inclusion criteria	Sample	Methods	Theoretical framework	Setting and intervention
Abel et al., 2018, New Zealand [34]	Exploring the barriers and facilitators of making dietary improvements among participants following a dietary intervention	Adults < 70 years with newly diagnosed prediabetes (HbaA1c 41–49 mmol/mol 5.9–6.6%), BMI ≥ 25 kg/m2 and metformin not prescribed	*n* = 20 F = 10 M = 10 Age range: 43–69 years	Semi-structured qualitative interviews, thematic analysis	Not described	Setting: A six-month primary care nurse-delivered dietary intervention pilot; Prediabetes Intervention Package (PIP) Area for lifestyle change (intervention): To promote dietary changes
Andersson et al., 2008, Sweden [35]	Exploring the experiences of individuals with pre-diabetes and the associated increased risk of type 2 diabetes	Prediabetic as described by WHO guidelines of 1998, including IGT/IFG -6 months after health check	*n* = 8 F = 5 M = 3 Age range: 30–74 years	Qualitative interviews Phenomenological hermeneutical approach	Not described	Setting: Health examination at the local health centre consisting of two visits, aim being to collect information on the general health and living conditions of the population of Skaraborg. The interviews were conducted after the health examinations Area for lifestyle change (no intervention): To promote exercise and dietary changes
Coppell et al., 2017, New Zealand [36]	Examining the implementation and feasibility of a six-month multilevel primary care nurse-led prediabetes lifestyle intervention compared with current practice in patients with prediabetes	Adults aged ≤ 70 years, if women non-pregnant with newly diagnosed prediabetes; HbA1c 41–49 mmol or FGP 6.1–6.9 mmol/L, BMI > 25 kg/m2, and metformin not prescribed	*n* = 20* F = 10 M = 10 Age range: 49–65 years *A subsample of patients who had completed at six-months intervention were purposefully selected to ensure a range of demographic profiles and glycaemic outcomes	Mixed methods, convergent design, involving a 6-month pragmatic non-randomised pilot study with a qualitative process evaluation Semi-structured qualitative interviews, thematic analysis	Not described	Setting: A six-month primary care nursing-led dietary intervention for prediabetes Area for lifestyle change (intervention): To promote exercise and dietary changes
Dyer et al., 2020, USA [37]	Assessing the impact of gender-tailoring and modality choice on a diabetes prevention program (DPP) engagement among women veterans with prediabetes	Female veterans with prediabetes (HbA1c 5.7–6.4% in prior 12 months), who were overweight or obese (BMI ≥ 24 kg/m2)	*n* = 15 * F = 15 Mean age: 55.5 *A subsample of participants from a larger study on diabetes prevention. The subsample participants were selected by random. Interviews were conducted at early-implementation (in person modality = 6 and online modality = 4) and post-implementation follow-up (in person modality *n* = 6 and online modality = 6)	Mixed method study, qualitative semi-structured telephone interviews	Not described	Setting: Tailored diabetes prevention program (DPP) implemented in a large Veteran Affairs (VA) health-care system from 2016 to 2018. Area for lifestyle change (no intervention): To promote exercise and dietary changes
Hansen et al., 2010, Norway [38]	Identify factors that could have motivational significance for lifestyle change to facilitate the reduction of impaired glucose tolerance (IGT) and, consequently, the risk of type 2 diabetes	People with IGT according to WHO guidelines	*n* = 18 F = 14 M = 4 Age range: 33–69 years	Semi-structured interviews, content analysis method	Health Belief Model	Setting: An instructed, controlled, physical strength exercise program for four months at a fitness centre (2–3 times per week). Interviews were conducted after the intervention Area for lifestyle change (intervention): To promote exercise
Jallinoja et al., 2008, Finland [39]	Exploring whether the individual is seen as capable of autonomously seeking a healthier lifestyle or is dependent on external control and support	Individuals with increased risk of type 2 diabetes but not diagnosed with diabetes	*n* = 30 F = 17 M = 13 Age range: 52–65 years The focus groups consisted of three weight-reducers’ interview groups (5 women, 10 men) and three weight-gainers’ interview groups (12 women, 3 men)	Focus groups Discourse analysis	Not described	Setting: The participants had 1 ½ years earlier participated in a group-based counselling program: GOAL (Good Aging in Lahti Region), a type 2 diabetes prevention program (8-month duration) Area for lifestyle change (intervention): To promote exercise and dietary changes
Korkiangas et al., 2011 [40]	Describing motivators and barriers of exercise among adults with a high risk of type 2 diabetes	Individuals who had either scored 15 points or more or 12 points or more on the diabetes risk test with an increased risk of work disability or elevated FGT or IGT in an oral glucose tolerance test within the last 12 months	*n* = 74 F = 33 M = 41 Mean age: 49 years	Focus groups, inductive content analysis Video conferences and face-to-face groups (data obtained from taped video conferences only)	Not described	Setting: Six-month follow up study on the effectiveness and feasibility of activating counselling methods and video conferences in dietary group counselling Area for lifestyle change (intervention): To promote exercise
Kullgren et al., 2015, USA [41]	Examining the frequency of, facilitators of, and barriers to prevent type 2 diabetes among employees found to have pre-diabetes during a workplace screening	Individuals who had FBG measurements of 100 to 125 mg/dl	*n* = 40* F = 29 M = 11 Age range: 41–57 *A subsample of participants from a larger study on diabetes prevention. The subsample participants were selected by purposive sampling with regard to whether they followed the recommendations or not	Mixed methods observational study Semi-structured telephone interviews	Not described	Setting: Follow up study on university employees (*n* = 82) who were found to have prediabetes during a workplace screening. After three months two groups were compared: 1) Participants attempting weight loss who have gotten at least 150 min of moderate physical activity since the screening or participating in a DPP or 2) have not carried out any of these recommendations after screening Area for lifestyle change (no intervention): To promote exercise and dietary changes
Kuo et al., 2013, Taiwan [42]	Exploring the experiences of people with prediabetes in relation to their engagement in exercise	Adults over 18 years with IFG, experience with exercise	*n* = 20 F = 11 M = 9 Age range: 38–66 Mean age: 52.3	In-depth semi-structured interviews Grounded theory	Not described	Participants were interviewed after health consultations Area for lifestyle change (no intervention): To promote exercise
Lim et al., 2020, Singapore [43] and 2019 [44]	Publication from 2020:Assessing factors associated with meeting the recommendation of at least 150 min of moderate/vigorous physical activity weekly and exploring facilitators and barriers related to the exercise behaviour among primary care patients with prediabetes in Singapore Publication from 2019: Assessing factors associated with fulfilling the healthy plate recommendation and exploring reasons for the dietary behaviour among primary care patients with prediabetes in Singapore	Community dwelling patients aged 21–79 years with existing prediabetes, diagnosis verified by oral glucose tolerance test (OGTT) and diagnosis code, and currently following up at any of the eight polyclinics	*n* = 48* F = 24 M = 24 Age range: 21–79 years *A subsample of participants from a larger study on diabetes prevention. Maximum variation sampling strategy was used to recruit a purposive subsample of participants from diverse backgrounds, based on the criteria of sex and whether they reported meeting the “My healthy Plate” recommendation	Mixed methods In depth interviews, thematic data analysis (Braun and Clarke)	Social ecological model (SEM) framework	Setting: Recruited from nine NHG (National Health Care Group) polyclinics in Singapore, health consultations Area for lifestyle change (no intervention): To promote exercise [43] and dietary changes [44]
Mayega et al., 2014, Uganda/ Sweden [45]	Assessing perceptions about type 2 diabetes and lifestyle change among people afflicted with or at high risk of the disease in a low-income setting in Iganga, Uganda	Three glycaemic categories: Suspected diabetes type 2: FPG as ≥ 7.0 mmol/l, suspected prediabetes as an FPG of 6.1–6.9 mmol/l and obesity as BMI > 30 kg/m^2^	*n* = 96 F = 47 M = 47 Age 35–60 years	Focus group discussions, content analysis Ethnographic approach	Not described	Setting: The study was conducted in the Iganga-Mayuge Health and Demographic Surveillance Site (HDSS) in eastern Uganda Area for lifestyle change (no intervention): To promote exercise and dietary changes
Morrison et al., 2014, Scotland [46]	Exploring the reasons for enrolling in, experiences participating in, and reasons for remaining in a family-based, cluster randomized controlled trial of a dietitian-delivered lifestyle modification intervention aiming to reduce obesity in South Asians at high risk of developing diabetes	Waist size ≥ 90 cm for men and ≥ 80 cm for women; IGT (i.e., fasting plasma glucose of < 7 mmol/l and, following a standard OGTT, a 2 h plasma glucose of 7.8–11.0 mmol/l) IFG (i.e., plasma fasting glucose of 6.1–6.9 mmol/l); no previous diagnosis of diabetes	*n* = 20* F = 7 M = 13 Age not described *A subsample of participants from a larger study on diabetes prevention. The subsample participants were selected by purposive sampling, ensuring diversity, within the trial population by sex, ethnicity, faith, group, geographical location (Glasgow and Edinburgh) and whether they were allocated to the intervention or control group	Narrative interviews, thematic analysis	Not described	Setting: A complex dietitian-led dietary-based and physical activity-based intervention for reducing obesity and preventing type 2 diabetes mellitus in people of Indian and Pakistani origin at a high risk of developing diabetes living in Scotland over a three-year period Area for lifestyle change (intervention): To promote exercise and dietary changes
Penn et al., 2008, England [47]	Understanding the experience of participants who maintained behaviour change aiming to inform future interventions. Exploring the dimensions of achieving and maintaining lifestyle change	At trial, first recruit: > 40 years, Caucasians with BMI > 25 and IGT diagnosed based on two OGTTs	*n* = 15 F = 7 M = 8 Age range: 47–74	Semi-structured interviews, content analysis, framework approach Empirical phenomenology approach	Not described	Setting: A study nested from the European Diabetes Prevention Study (EDIPS); intervention was individual motivational interviewing (three-month interval) aiming to reduce total food energy and fat intake and increasing activity. Exploring experiences of the participants 3–5 years after Area for lifestyle change (intervention): To promote exercise and dietary changes
Strachan et al., 2018, Canada [48]	Exploring how people from a small Canadian city diagnosed with prediabetes react emotionally to their diagnosis	Between 18 and 65 years old, diagnosed with prediabetes according to the ADA (2016): HbA1c = 5.7%-6.4% (*n* = 20) or ADA risk-questionnaire indicating increased risk (> 5 = 1) within the past year	*n* = 21* F = 18 M = 3 Age range: 47–65 years *A subsample of participants from a larger study on diabetes prevention. The subsample participants were selected by random	Semi-structured interviews, inductive thematic analysis (Braun and Clarke)	Not described	Setting: The study was part of a larger project where people with prediabetes participated in a 3‐week community‐based lifestyle intervention in Western Canada, “Small Steps for Big Changes Program (SSB)”. Participants were interviewed prior to involvement in the intervention Area for lifestyle change (intervention): To promote exercise and dietary changes
Bean et al., 2020, Canada [49]	Two-fold purpose of the study: a) Profiling patterns of women’s perceived PA journey over one year in those who engaged in Small Steps for Big Changes (SSBC) and b) understanding strategies used to engage in and maintain PA	Participants were a) between 18- and 65-years old b) able to read and speak English, c) identify as a woman, d) have prediabetes (glycated haemoglobin 5.7% to 6.4%), and e) have completed SSBC	*n* = 14* F = 14 Age range: 48–63 Mean age: 60.07 *A subsample of participants from a larger study on diabetes prevention who were female and eligible for SSBC participation, were invited to participate in the qualitative sub study	Qualitative semi-structured interviews, face-to-face and telephone interviews Trajectory approach coupled with a deductive-inductive thematic analysis (Braun and Clarke)	Not described	Setting: The study is a follow-up to a three-week community-based diabetes prevention program in Canada; Small Steps for Big Changes (SSBC) Participants were interviewed at baseline, during and after the intervention Area for lifestyle change (intervention): To promote exercise and dietary changes
Katangwe et al., 2020, England [50]	Exploring factors influencing engagement with the National Health Service (NHS) DPP and the role of community pharmacies (CP) in diabetes prevention	Eligible patients for referral: individuals 18 years or over with HbA1c blood test results within the pre-diabetes range (42–47 mmol/mol [6.0–6.4%]) in the last 12 months	*n* = 16* F = 9 M = 7 Mean age: 68.4 *A subsample of participants from a larger study on diabetes prevention were purposively sampled from the questionnaire respondents for follow-up semi-structured interviews (*n* = 10) and a focus group (*n* = 6)	Explanatory sequential mixed method design Focus groups and semi-structured interviews (telephone) Thematic analysis (Braun and Clarke)	COM- B approach, theoretical model for identifying key factors influencing desired behaviours	Setting: Individuals were invited to participate in the NHS DPP in order to lower their risk of developing T2D. In the interview study sample, three had attended the program, three had completed the program, three were waiting, two had dropped out, and five had declined Area for lifestyle change (intervention): To promote exercise and dietary changes
Griauzde et al., 2020, USA [51]	1) Estimating weight change from a low-carbohydrate diabetes prevention programme (LC-DPP) and 2) evaluating the feasibility and acceptability of a LC-DPP. General experiences with the intervention as well as specific barriers and facilitators of VLCD adherence were specifically explored	(1) Overweight (BMI ≥ 25 kg/m2), (2) haemoglobin A1c (HbA1c) between 5.7% and 6.4% drawn within six months of the study start date, (3) willingness to participate in group-base class, and (4) ability to engage in at least light physical activity	*n* = 14* F = 8 M = 6 Mean age: 58.7 *A subsample of participants was recruited from the dietary intervention, sampling not described	Mixed methods sequential explanatory study design Single arm pilot study Qualitative semi-structured interviews	Not described	Setting: Primary care clinic within a large academic medical centre in the USA. An evidence based, low-fat dietary intervention to teach participants to follow a very low carbohydrate diet (VLCD). Participants attended 23 group-based classes over one year. The participants were interviewed at six (n = 13) and 12 months (*n* = 12) Area for lifestyle change (intervention): To promote dietary changes
Howells et al., 2021, England [52]	Exploring how individuals with prediabetes understand biomedical definitions of risk and the extent to which they resist them, as this reframing of risk could have implications for engagement with the NHS DPP	High-risk patients defined as prediabetic via a blood glucose test (HbA1c level 42–47 mmol/mol) and who had received their prediabetes diagnosis within the last 12 months	*n* = 43* F = 20 M = 23 Mean age: 60 *Seven general practices were purposively selected to recruit participants (based on a range of factors, including deprivation scores, ethnic diversity and their approach to informing their patients about diabetes risk). From these, all eligible at-risk patients were invited to have consultations audio-recorded and to also participate in in-depth interviews	Qualitative mixed methods: observational study (audio recorded consultations), and individual in-depth interviews Grounded theory approach	Not described	Setting: In the context of a national diabetes prevention program, the setting of this study is the primary care consultation where data was drawn from individual interviews and observations Area for lifestyle change (no intervention): To promote exercise and dietary changes
Wallace et al., 2021, USA [53]	Understanding how Latinos with prediabetes attempted to slow T2D progression and how stress affected their engagement in these behaviours	(1) 20 years of age or older; (2) born in a Spanish-speaking Latin American or Caribbean country; 3) doctor confirmed prediabetes diagnosis (haemoglobin A1c range 5.7–6.4%) or elevated fasting glucose reading 100–125 mg/dL in past year, and (4) received medical care in the health system in the past year	*n* = 20 F = 14 M = 6 Age range: 22–72 Mean age: 51	Qualitative semi-structured interviews	Not described	Setting: Participants were interviewed after having been diagnosed with prediabetes, following participants’ medical appointments Area for lifestyle change (intervention): To promote exercise and dietary changes

* How participants in the qualitative data collection were selected if part of a larger study

*IFG* Impaired fasting glucose, *IGT* Impaired glucose tolerance, *FPG* Fasting plasma glucose, *FBG* Fasting blood glucose, *OGTT* Oral glucose tolerance test, *BMI* Body mass index, *T2D* Type 2 diabetes and *DPP* Diabetes prevention program

*F* Female, *M* Male

Thirteen studies reported on the participant perceived facilitators and barriers of lifestyle change when taking part in community-based lifestyle intervention programs [34, 36–40, 46–51, 53], while seven studies reported on the participants perceived facilitators and barriers of lifestyle change through consultations with health care providers (no intervention involved) [35, 41–45, 52]. Thirteen studies [35–37, 39, 41, 45–50, 52, 53] reported on the barriers and facilitators of lifestyle change and behavioural change maintenance, addressing both exercise and diet (participants exposed to an lifestyle intervention in nine studies, whereas no intervention in four studies), four studies [38, 40, 42, 43] reported on exercise only (participants exposed to an lifestyle intervention in two studies, whereas no intervention in two), and three studies [34, 44, 51] reported on diet only (participants exposed to an lifestyle intervention in two studies, whereas no intervention in one).

### Quality assessment

Of the then criteria used to assess the methodological quality [33], all the included studies met seven or more of these criteria. Two studies [36, 51] were graded with seven out of ten points, three studies [37, 38, 53] were graded with eight points, six studies [39, 41, 45–47, 52] were graded with nine points and nine studies [34, 35, 40, 42–44, 48–50] with ten points (Table 2). The relationship between the researcher and participants were one domain that was assessed not to be adequately described in several of the included studies [37–39, 41, 45, 47, 51–53].

### Thematic synthesis of the qualitative studies

**Fig.2:**
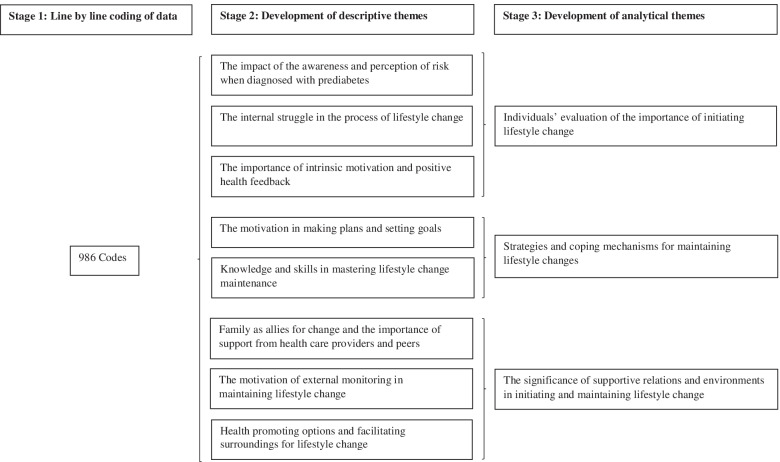
Emergent descriptive and analytical themes

In total 986 codes were recorded from the extracted data, from which eight descriptive themes emerged. From the synthesis and analysis of the included primary studies, three main themes illuminating the perceived barriers and facilitators of lifestyle change among people with prediabetes were identified: 1) the individual’s evaluation of the importance of initiating lifestyle change; 2) strategies and coping mechanisms for maintaining lifestyle change; and 3) the significance of supportive relations and environments in initiating and maintaining lifestyle change (Fig. 2).

In general, the primary studies demonstrated that there are multiple barriers and facilitators in the process of lifestyle change, and they exist in a complex interplay. Table 4 presents how the different primary studies are distributed across the main themes and subthemes based on whether they included lifestyle intervention programs or not, and the area of lifestyle change, being exercise or diet, or both. The presentation of the results is supplemented with quotes from participants in the included primary studies.

**Table 4:** Cross-tabulation of themes and sub-themes by intervention and area for change

		Intervention (structured lifestyle intervention program)			No intervention			
**Main theme**	**Subthemes**	**Exercise and diet**	**Exercise**	**Diet**	**Exercise and diet**	**Exercise**	**Diet**	**Number of studies**
Individuals’ evaluation of the importance of initiating lifestyle change	The impact of the awareness and perception of risk when diagnosed with prediabetes	[36, 39, 46–48, 50]	[38]	[34]	[35, 41, 45, 52]	[42, 43]	[4]	15
	The internal struggle in the process of lifestyle change	[36, 39, 46–50]	[38, 40]	[34, 51]	[35, 41, 45, 52]	[42, 43]	[44]	18
	The importance of internal motivation and positive health feedback	[36, 46, 47, 49, 53]	[38]	[34, 51]	[41]	[42, 43]	[44]	12
Strategies and coping mechanisms for maintaining lifestyle changes	The motivation in making plans and setting goals	[36, 37, 39, 46, 47, 49, 50, 53]	[38]	[34, 51]	[35, 41]	[43]	[44]	15
	Knowledge and skills in mastering lifestyle change maintenance	[36, 46, 47, 49, 50]	[38, 40]	[34, 51]	[35, 41, 45, 52]	[43]	[44]	15
The significance of supportive relations and environments in initiating and maintaining lifestyle change	Family as allies for change and the importance of support from health care providers and peers	[36, 46–50, 53]	38, 40]	[34, 51]	[35, 41, 45]	[42, 43]	[44]	17
	The motivation of external monitoring in maintaining lifestyle change	[36, 37, 39, 46, 47, 49, 50, 53]	[40]	[34, 51]	[35, 41]		[44]	14
	Health promoting options and facilitating surroundings for lifestyle change	[36, 39, 47–50, 53, 37]	[40]	[34]	[35, 41]	[43]	[44]	14

#### Theme 1: The individual’s evaluation of the importance of initiating lifestyle change

The first theme focused on the impact of the awareness and perception of risk on the individual’s evaluation of the importance of initiating lifestyle change, specifically considering reactions to the diagnosis of prediabetes and the internal struggle during the process of lifestyle change.

#### The impact of the awareness and perception of risk when diagnosed with prediabetes

 Our analysis revealed that a vital facilitator in healthy lifestyle changes was when people became aware of being at a high risk of developing type 2 diabetes and realized the potential threat to their health. They experienced fear regarding the consequences of disease and facing an uncertain future [34, 36, 38, 41–48, 50, 52, 53]. Several participants in the primary studies reflected on the experience of having family members diagnosed with diabetes and expressed the desire to stay healthy and alive for their children and grandchildren to not become a burden to their family [34, 36, 38, 41, 43, 44, 46–48]. For example, one individual said:

There’s a big element of worry . . . like I’m on the train and I can’t stop it. You get that worry of ‘are you going to be able to stop this from getting worse?’ . . . like ‘whoa, what’s going on here?’ . . . I don’t want to become diabetic, that would be my main concern, I don’t want what comes with that. [48]

Several participants in the reviewed studies were aware of the increased risk of the progression to type 2 diabetes if lifestyle changes were not made and they were determined to stay ahead of their disease development [34, 36, 38, 41–44, 46–48, 50, 53]. In one of the included studies, participants reported that, at the time of their prediabetes diagnosis, their health care consultations provided little to no information on how to comprehend and understand the impact of its risk [52]. Several participants described shock when diagnosed with prediabetes [34, 36, 38, 49, 50, 52, 53]. For some participants this shock motivated them for lifestyle change, others found it difficult to identify themselves as being in an ‘at risk state’, as this conflicted with their own perceptions of having a healthy lifestyle creating a distance to future risk [42, 45, 48, 52, 53]. Hence, the findings illustrated how the recognition of prediabetes as asymptomatic and not associated with a medical condition or equated with severe illness led to a downplaying of the risk by the participants in the reviewed studies [42, 45, 48, 52, 53].

#### The internal struggle in the process of lifestyle change

 Feelings of both guilt and self-blame arose with a diagnosis of prediabetes. The findings illustrated this phenomenon by describing how participants in our included studies accepted a personal responsibility for their outcomes [34, 35, 38, 39, 42, 47, 48, 50–53]. In one study, a participant expressed a sense of commitment and personal responsibility to society in terms of lifestyle change and preventive behaviours [50]. Internal struggles with self-criticism and self-blame, especially when it came to dietary changes, were described by several participants in the included studies as leading to lower self-esteem and a lack of confidence, which, in turn, inhibited the driving force for change [35, 39, 42, 47, 48, 53]. An individual described this feeling in the following way:

How am I going to do this? It seems so overwhelming. I know I should ideally lose a hundred pounds to get back to…my ideal weight, but it seems like such an insurmountable mountain to climb that why even try? [48]

A recurrent theme in our findings was how the gap between behavioural intentions and actual behaviour change amplified the negative feelings of guilt and self-blame that, in turn, lead to stress [34, 35, 39, 48, 52, 53]. One of the studies demonstrated that stress affected behaviour change in terms of different emotional and cognitive responses for the participants in the included studies, with participants describing how this challenged their self-control, decision-making, and self-regulation [53]. One participant stated:

Sometimes I get very angry at myself because I don’t have the self-control to say: ‘stop eating that and go and exercise.’ Typically, I intend to do it, but then I feel anxious and I go and eat a pastry or something like that. Then after I feel terrible and I start thinking, how is it possible that I cannot get over this stress? [53]

Several of the studies described how temptation for sweet foods challenged the participants’ sense of self-control, making it difficult for them to implement healthy changes in their diet [34, 36, 44, 45, 47, 51–53]. One study described how increased awareness regarding the necessity of dietary change created new cravings and temptations [53]. For some participants, having to reduce sugar and missing the sweet taste of foods were particularly challenging [34, 36, 45, 48, 51, 53], describing it as a feeling of sacrificing the good life [45].

In some studies, the participants described that the stress and energy involved in making lifestyle changes would compromise their quality of life, also noting that they had greater concerns than progressing to diabetes [34, 39, 40, 42, 47, 48, 52, 53]. One participant expressed the following:

I think there’s always a risk, I think there’s always some sort of risk, but it’s a very . . . I put it really on the backburner. If you think of priorities, it’s falling downstairs or tripping over, and I do try and eliminate risk. This is why I’ve started off with this Pilates teacher, which is definitely making me more aware of balance. Diabetes, it doesn’t worry me particularly. [52]

#### The importance of internal motivation and positive health feedback

 Our findings demonstrated that experienced positive health feedback among the participants facilitated lifestyle change. For example, participants from several of the studies experienced benefits from exercising, such as improved physical condition and mental well-being. This encouraged them and led to a sense of accomplishment [41, 43, 47, 49, 53]. Improved physical condition, mental well-being, the enjoyment of different activities, and taking pleasure in nature were described as drivers of the maintenance of exercise change [38, 40, 41, 43, 46, 47, 49, 52, 53]. This sense of overall well-being and enjoyment was depicted as a central autonomous motivation for exercise, and, for many participants, exercise was also connected with being outdoors and taking pleasure in nature [38, 40, 41, 43, 49]. Accordingly, one individual described the following:

So, when you go outside to exercise, you feel the sunshine, you breathe in the fresh air, your body will then be good. It is for our wellbeing. [43]

Several participants in the included studies highlighted the value of former experience with exercise and how this facilitated their self-confidence to seek new activities that gave them further positive experiences with exercise [40, 42, 43, 46, 49, 52, 53]. Some participants explained that exercise also became integrated into their sense of self when it became a routine and a habit. Being able to identify oneself as a person with an active lifestyle and the desire to be a good role model for one’s children were facilitators for lifestyle change [38, 40, 47, 49]. Participants also reported experiencing a sense of self-control that strengthened their motivation to adhere to a regular exercise regimen [43, 46, 50, 51].

As with exercise, receiving positive health feedback from dietary change was described as giving a sense of mastery and self-control that facilitated maintenance. The participants in some of the studies experienced weight loss, a decrease in blood pressure, and a reduction in medication use in terms of dosage, as well as increased energy and improved sleep [34, 42, 44, 46, 51, 53].

##### Theme 2: Strategies and coping mechanisms for maintaining lifestyle change

The focus in the second theme was on the strategies and coping mechanisms involved in lifestyle change maintenance, including making plans and setting attainable goals and the importance of knowledge and skills in mastering lifestyle change maintenance.

###### The motivation in making plans and setting goals

Making plans and setting goals were helpful facilitators of initiating and maintaining lifestyle change. Several studies emphasized that the process of guiding one’s own thoughts, behaviours, and feelings was important in order to make more concrete plans and set realistic and specific goals [34, 36, 38–41, 47, 49, 51, 53]. One participant noted:

I established a goal. I force myself to run three laps no matter how sluggish I feel. . . If I run today, I feel that I have paid attention to my health and I feel at peace. [39]

In two of the studies, self-compassion was highlighted as a strategy for making plans and setting goals [48, 49]. Being kind to oneself was also put forward as making it easier to set attainable goals and prioritize oneself in finding the space, energy, and time for healthy changes [48, 49, 52, 53]. Making time for lifestyle change was presented as a challenge in the process of making plans and reaching goals. Obligations regarding time, such as family commitments and workload, were often mentioned as barriers to participants being more physically active [34, 38, 40–43, 46, 47, 49–51, 53]. In several studies, female participants described how they found it difficult to find the time for and prioritize exercise when fulfilling their various responsibilities as wives, mothers, daughters, and, in some cases, caregivers [42, 43, 46, 47, 51, 53]. One participant described their obligations as follows:

From Monday to Friday, I’m working . . . then Saturday and weekend I need to run errands for my children, my husband, and on top of that there is the housework. I also need to spend some time to visit my parents. Time is very important to me, I have so many duties and roles to fulfil, my first priority is always my family. [43]

Male participants, however, more often cited work as their reason for having ‘no time’ [43]. For example, one explained:

I am always so busy . . . in the evenings there are always papers to look at, I have no time for exercise. . . I simply don’t have the time. [41]

#### Knowledge and skills in mastering lifestyle change maintenance

The included studies presented a broad range of accounts about how one strategy for coping with lifestyle changes involves attaining knowledge, competence, and skills regarding exercise and a healthy diet for managing change [34–36, 39, 41, 44, 46, 47, 49–53]. Some of the studies demonstrated how knowledge and understanding affected how the participants behaved, enabling them to re-evaluate former habits [35, 41, 43, 44, 49, 51, 52].

The importance of skills and competence was highlighted in our included studies [43, 44, 46, 47, 49, 51, 53], with one woman describing the following:

. . . my cooking is all standard, you add the oil, the salt, and the sauce. But if you ask me to cook healthy food, like reduce the oil, reduce the salt, don’t use the sauce, then I don’t know how to cook already. Also, I have been cooking white rice all my life, now you tell me change to brown or red rice, I don’t know how to cook, how to make it tasty like white rice. [44]

Health care providers can help people with prediabetes by supplying them with information and guidance that will equip them with the knowledge, competence, and skills they need to facilitate and manage lifestyle changes and the risk they are facing [34, 36, 37, 41, 42, 46, 47, 49, 50]. Specifically, one participant mentioned the following:

It wasn’t stop this, stop that. It was cut down on this, cut down, little steps. . .The favourite saying is ‘little steps.’ And that’s probably one of the most helpful sayings I’ve ever heard. Not trying to do it in a week or two weeks, or two months or three months. It’s over a period of time, you know? [34]

Because of the perceived complexity of information regarding lifestyle change, several participants emphasized the importance of clarity and simplicity as well as pedagogical and empowering dialogue [34–37, 41, 46, 47, 49, 50]. Access to information and guidance in developing manageable strategies were also deemed vital for coping with lifestyle changes [34–37, 41, 46, 47, 49, 50].

#### Theme 3: The significance of supportive relations and environments in initiating and maintaining lifestyle change

The third theme focuses on the role of supportive relations being support from family, health care providers and peers in initiating and maintaining lifestyle change. In this final theme, supportive environments include external monitoring and support from lifestyle intervention programs, facilitating surroundings, and the availability of health promoting options for lifestyle change.

#### Family as allies for change and the importance of support from health care providers and peers

In the included studies, the spouse or children of the participants were described as important allies when it came to motivation for initiating and continuing lifestyle changes. Several participants highlighted how support from family members acted as a form of supervision, with family members checking up on them and encouraging shared decisions in facilitating healthy behaviours [40, 42, 49, 51, 53]. In terms of making dietary changes, the influence of one’s spouse and children was also noted as playing an important role in whether recommendations from health care providers were met or not. This influence could take the form of informative reminders from family members in meal situations [34, 36, 44, 47, 51, 53]. For example, one woman mentioned:

My children will say, ‘mom that’s salty, don’t eat’ or you know, they will say ‘this is too fat, don’t eat’, you know what I mean? They will remind me and keep a look-out on my diet. [44]

Acceptance of the necessity of change within the family was another important factor for participants. A mutual understanding of the process of change was described as leading to increased involvement and support from family members, which, in turn motivated and encouraged participants [34, 36, 42, 44, 51, 53]. Some studies also pointed out that family norms regarding being active could be part of participants’ identities and family cultures. In our findings, this was demonstrated to facilitate attempts to make lifestyle changes [41, 43, 51, 53]. On the other hand, family norms, traditions, and culture could sometimes be barriers to lifestyle change, especially in terms of dietary changes [34, 36, 44–46, 51, 53]. The studies found that the participants described social expectations and pressure around providing and being offered foods as a challenge, with family gatherings and parties presented as examples of challenging settings with fewer healthy food options [34, 36, 44–46, 51, 53]. In the context of everyday life, food traditions and eating norms in families could also sometimes make dietary change difficult [34, 36, 44, 51, 53]. One individual described the following:

My whole family eats white rice since young, it has become a habit, a culture in us. Now say change to brown rice, not easy, it takes time for us to adjust to the new taste of brown rice. [44]

Receiving support and encouragement and not feeling alone in making lifestyle changes were described as positive effects of joining a group with other people with prediabetes [34, 36, 37, 39, 41, 48, 50]. Participants specifically described the benefits of sharing their experiences, exchanging ideas and strategies, and being motivated by each other [34, 36, 37, 39, 41, 48–50]. Some participants highlighted that, when participating in a lifestyle program and joining a group with peers, external support from peers led to more physical activity and exercise on their part [39, 41]. In one study, female participants described the importance of support from other women in a female-only setting, emphasizing the mutual understanding of barriers and other experiences that are specific to women [37].

Empowering communication was highlighted by participants in all studies as a key factor facilitating the supportive function of health care providers [34, 36, 37, 39, 41, 44, 46, 47, 49, 51]. Participants in most of the studies emphasized how health care providers could facilitate lifestyle change [34, 36, 37, 39, 41, 44, 46, 47, 51]. Feeling accountable, receiving trusted communication and care, and being addressed with respect and empathy were also identified as important characteristics of this support [34, 36–38, 41, 44, 46, 47]. One woman, when explaining how her health care professional helped her, stated the following:

It was the way she encouraged me, how she uplifted me. I am so grateful . . . So, I think having the right people at the forefront there just to open you up, you know, and acknowledging where I am at. [34]

#### The motivation of external monitoring in maintaining lifestyle change

 In several studies, the participants highlighted that a successful facilitator they strongly valued was being monitored in intervention programs during the process of lifestyle change [34, 36–40, 46, 47, 49, 50]. Participating in a program imparted a sense of commitment on them, and the participants were held accountable for their attempts to make healthy changes [34, 36–40, 46, 47, 49, 50]. Having to report on their progress to a supervisor or having official measurements of their weight loss or improved physical condition taken in the near future, were described as strong motivators encouraging the participants to push themselves [36–38, 40, 46, 47]. Tailoring lifestyle interventions to individuals also seemed to facilitate the process of making healthy changes. The freedom of choice and flexibility in a tailored program was seen to allow participants to set personalized and meaningful goals [34, 37, 46, 47, 50].

Five studies highlighted the importance of technological devices in monitoring healthy lifestyle change and how such devices could provide support for those not participating in a lifestyle intervention program. The data from step-counter technology and the feedback provided from this was described as motivating and inspiring [37, 41, 49, 53]. For example, a user of a Fitbit stated:

I have a Fitbit that makes it easier, because I like to challenge myself to make sure I get my steps every day. So, lots of times, I’ll get home in the evening and I’ll see them at 9000 steps, and I’ll like go out and walk up and down the driveway. [41]

The value of using digital tracking and apps to document the process of change and regulate food consumption was also described as an external motivation in terms of dietary change [53], with one participant expressing the following:

I must not just settle with reducing carbohydrates, but I must, as we say, document it. I had a friend that believed that, for everything you did, you had to keep a record of it and said, ‘It’s like sports; if you don’t keep a record, you’re only practicing. [53]

In a study that used an online-modality lifestyle intervention program, the participants highlighted the logistical benefits of the flexibility and convenience of a digital follow-up [37], showing how this could make lifestyle intervention programs more accessible regarding distance and geography or according to work schedule or family obligations.

#### The availability of health promoting options and facilitating surroundings

 Participants described experiencing barriers and facilitators of lifestyle change in their work environments, in their neighbourhoods, in their local communities, and at the societal level [34, 38, 40–47, 49–51, 53]. For example, three of the studies described how making healthy changes to one’s diet was challenging when there were limited healthy options at the workplace or local restaurants [34, 44]. Several participants cited financial restraints as barriers to lifestyle change [34, 36, 44, 45, 47, 51, 53], with the high cost of healthy food leading some to choose unhealthy food because it was the more affordable option [34, 36, 44, 45, 47, 51, 53]. For example, one individual stated:

Look, the barrier to those goal settings is budget, you know . . . So, when you see on TV people saying they’re eating unhealthily, what they’re doing, what we’re doing is we’re eating to a budget planned to survive for the week.... So, don’t go telling poor people ‘you’re going to get diabetes if you eat this and this and this’; so we want you to eat this food, but it’s too expensive for you to buy, you know. [36]

In several studies, we found that having access to exercise facilities and organized activities in local communities, parks, and green areas made it easier to initiate and maintain physical activity and exercise [35, 38, 40, 41, 43, 46, 47, 49]. However, climate and weather conditions could affect access to those spaces and some participants experienced bad weather and climate as a barrier to exercise [38, 40, 41, 43, 46]. Having access to nature and outdoor life was also described as an important facilitator for physical activity [41, 43, 49]. Moreover, some participants pointed out that it was too expensive for them to use indoor training facilities. In one study, participants acknowledged a governmental health promotion strategy to lower the cost of accessing different indoor training facilities as a positive solution [47].

## Discussion

This meta-synthesis aimed to explore, synthesize, and interpret qualitative research on facilitators and barriers of lifestyle change and maintenance among people with prediabetes. In line with the ecological framework, our findings indicate that the relevant barriers and facilitators are found within the intrapersonal, interpersonal, environmental, and policy level. We identified three main themes within these ecological levels being the individual’s evaluation of the importance of lifestyle change, strategies and coping mechanisms for maintaining lifestyle change and the importance of supportive relations and environments in initiating and maintaining lifestyle change. These themes are not independent, they exist in a complex interplay, which our discussion will reflect. In addition to the ecological framework [17, 21] the findings will be discussed in light of the central themes in the theoretical explanations of behavioural change maintenance presented in the review by Kwasnicka et al. [15].

### The individual’s evaluation of the importance of initiating lifestyle change

At the intrapersonal level, individual motives are crucial for initiating and maintaining behaviour change and are the drivers of volitional behaviour [15]. Our findings indicate that getting the diagnosis of prediabetes, affected the participants’ perception of risk and motivation towards initiating lifestyle change, but the internal struggle experienced by many participants also affected the individual’s evaluation of the importance of initiating lifestyle change. These findings align with the review by Kwasnicka et al. [15] in highlighting the importance of intrinsic motivation and autonomy in facilitating the maintenance of initial lifestyle change.

#### The impact of the awareness and perception of risk when diagnosed with prediabetes

Using the label ‘prediabetes’ on individuals at high risk of type 2 diabetes may increase the perceived threat of developing diabetes [55]. Our findings illustrate that the recognition of prediabetes as asymptomatic and not equating it with severe illness in some cases led to a downplaying of the associated risk [48, 52, 53]. This reveals some of the complexity of initiating lifestyle change in the face of an invisible disease; thus, this is perhaps what sets the prediabetes population apart from other high-risk populations. Our findings and previous research [23, 24] suggest that health care providers should emphasize illness severity and provide cues to action to encourage health behaviours, whilst at the same time acknowledging the fear and insecurity that might arise when dealing with the diagnosis of prediabetes.

#### The internal struggle in the process of lifestyle change

According to a systematic review and meta-analysis by Hennessey et al. [58], struggle in the process of lifestyle change may create stress and deplete one’s cognitive and emotional capacity, which, in turn, challenges or disrupts the self-regulatory capacity. Kwasnicka et al. [15] state that self-regulation is a limited resource, and coping with behavioural barriers, overcoming temptations, managing lapses, and avoiding relapses is a demanding process and requires sustained effort. This might explain why participants in the included studies searched for a balance between preserving their mental needs and focusing on preventive behaviours [34, 39, 40, 42, 47, 48, 52, 53]. According to Kwasnicka et al. [15] individuals are more likely to initiate behaviour change at times when their psychological and physical resources are plentiful, and the opportunity costs are low. Our findings reflected that when resources are low, individuals need more guidance and support in order to cope with the initiation and maintenance of lifestyle changes, especially when it comes to setting attainable goals and maintaining a balanced effort in everyday life.

#### The importance of intrinsic motivation and positive health feedback

According to the review by Kwasnicka et al. [15], the motivation to avoid negative health consequences is hypothesized to be insufficient to maintain preventive behaviour requiring maintained effort. In line with our findings, individuals are intrinsically motivated when lifestyle change is perceived as personally relevant and resembling one’s values and beliefs [16]. To support individuals with prediabetes in the process of initiating and maintaining lifestyle change, as well as to enhance intrinsic and autonomous motivation, it seems important that health care providers explore the individual’s perceptions of risk, their beliefs, and their personal values. In line with the ecological model this also pertains to the individual differences in culture and their different social and environmental contexts [21].

Several participants in the included studies experienced success with exercise and dietary changes after lifestyle change interventions. This was experienced through perceived positive health feedback, such as improved physical condition, weight loss, and this enhanced self-efficacy in the participants [41, 43, 47, 49, 52, 53]. The attainment of prior success and one’s own perception of a positive psychological state are, according to Bandura [59], suggested to increase self-efficacy and are therefore important for behavioural change maintenance. This is in line with Rothman [60], who emphasizes that the individual’s decision to maintain a behaviour change is dependent on their perceived satisfaction with the received outcomes.

### Strategies and coping mechanisms for maintaining lifestyle change

The process of making plans and setting goals, knowledge and skills and the formation of habits, are important aspects in the process of identifying strategies and coping mechanisms to maintain lifestyle changes [16]. These aspects are discussed mainly at the intrapersonal level but they cannot be understood isolated from social, environmental, and societal influences.

#### The motivation in making plans and setting goals

According to Hennessy et al. [58], setting goals initiates self-regulation and acts as a key mechanism for behaviour change. Self-regulation refers to any effort to actively control unwanted behaviour by inhibiting dominant and automatic behaviours, such as urges, emotions, or desires, and replacing them with goal-directed responses [15]. A systematic review by Leman et al. [61] found that people require self-efficacy and self-regulation to motivate their consistent performance of healthy behaviour.

Several participants in the included studies experienced a gap between their behavioural intentions and actual behaviour change, which then amplified their feelings of self-blame, guilt, and shame, especially when in terms of dietary changes [34, 35, 48, 52, 53]. This can cause dissatisfaction and lead individuals to either expend greater effort toward achieving the lifestyle change goals or disengage from these goals [15]. This underlines the importance of setting attainable, personal, relevant, and intrinsically motivated goals.

In two of the included studies, self-compassion was put forward as a strategy for making plans and setting goals [48, 49]. According to Neff [62], self-compassion entails three main overlapping and interacting components: self-kindness versus self-judgement, common humanity versus isolation, and mindfulness versus over-identification. Interestingly, in a recent meta-analysis by Liao et al. [63], a positive association was found between self-compassion and self-efficacy, indicating that self-compassion may play a role in protecting one’s self-efficacy when experiencing failures [63].

#### Knowledge and skills in mastering lifestyle change maintenance

A Finnish study of adults with increased risk of type 2 diabetes found that eating competence is associated with a healthy diet and could therefore, in the long term, support the prevention of type 2 diabetes [64]. Supporting autonomy and confidence is central in facilitating competence [16] and health care providers therefore play an important role when giving information and guidance. According to Gardner et al. [65], habit formation takes place after a period of the successful self-regulation of a new behaviour, and this is considered to play a fundamental role in generating health behaviour. Once a new behaviour has become a habit, it requires less effort, and the level of required self-regulation is reduced [15]. Gardner et al. [65] stated that habits persist even when conscious motivation decreases, and, therefore, habit formation should be encouraged in interventions to promote long-term maintenance.

### The importance of supportive relations and environments in initiating and maintaining lifestyle change

Within the ecological framework supportive relations and environments were identified at the interpersonal level, the environmental level and the policy level, affecting the motivation for initiating and maintaining lifestyle change for individuals with prediabetes.

#### Family as allies for change and the importance of support from health care providers and peers

At the interpersonal level of the ecological framework, supportive relations and social influence can be found in formal and informal social networks [21]. In line with the ecological perspective, Barry et al. [66] highlighted the importance of socio-cultural influences in diabetes prevention policies. When addressing barriers and facilitators for lifestyle change, we must consider the impact of social norms and cultural aspects within families and communities and consider how health behaviours are shaped within different contexts [67]. Considering this, lifestyle intervention programs and health care communication aiming to facilitate lifestyle change in people with prediabetes, should include and involve the families or other significant persons in the whole process. This could enhance the individuals’ perceived sense of relatedness in the lifestyle change process, which is important in maintaining a new behaviour [16]. In line with our findings, peer support can enhance the internalization and maintenance of lifestyle change through perceived relatedness, connection, and trust [16].

#### The motivation of external monitoring in maintaining lifestyle change

A systematic review and meta-analysis that investigated the best method to improve self-efficacy to promote lifestyle and recreational physical activity in healthy adults [68], found that interventions that included feedback on their past performance or others’ performance (comparative feedback) produced the highest levels of self-efficacy.

Lifestyle intervention programmes are not necessarily suitable for all individuals with prediabetes. This can be due to different life phases, family settings or personal preferences; or practical or logistical barriers, such as care responsibility, work, or geographical distance. In one study offering an online-modality lifestyle intervention programme, participants highlighted the logistical benefits of the flexibility and convenience of a digital follow-up [37]. There is promising evidence regarding the efficacy of diabetes prevention eHealth interventions [69], and the integration of specific behaviour change techniques and digital features may optimise digital diabetes prevention interventions achieving clinically significant weight loss in individuals with prediabetes [70]. At the same time our findings described that the use of technological devices and digital follow-up was motivating and inspiring [37, 41, 49, 53] and this further supports the potential of acceptance and increased use of digital eHealth interventions in the prevention of type 2 diabetes.

#### The availability of health promoting options and facilitating surroundings

In line with the ecological model and our findings, barriers and facilitators to promote healthy diet and physical activity in our external environment are to a great extent beyond the control of the individual. McLeroy [21] referred to “the ideology of individual responsibility” and how this may inhibit our understanding of the potential environmental assault on health and the opportunities for healthy behaviours. According to the review by Barry et al. [66], watchfulness should be put towards a biomedical approach where prediabetes is recognized as a reversible state of abnormal glucose metabolism that can be reversed solely by altering the individual patient’s lifestyle. This may lead to an overemphasis on the individual’s responsibility for lifestyle change, resulting in the creation of policy neglecting the complex sociocultural environment affecting health and illness. Therefore, identifying behaviour change and maintenance strategies that are tailored for individuals with prediabetes in their socio-cultural environment, is of great importance for the individual having prediabetes as well as for the society in order to reduce their risk of progression to type 2 diabetes [71].

At the public policy level, there are a range of incentives policy makers can use to influence health behaviour for the population and the individuals at risk for type 2 diabetes, including legislation, information campaigns and price signals [72]. A systematic review and meta-analysis has shown that the risk of being diagnosed with type 2 diabetes is associated with low socio-economic status [73]. Moreover, individuals of a lower level of socioeconomic status are more often exposed to negative lifestyle habits, such as smoking, physical inactivity, obesity, and low fruit and vegetable consumption [74]. Thus, a central challenge when implementing lifestyle interventions in practice is reaching people with prediabetes across social groups and socio-economic positions to avoid reinforcing health inequalities.

### Strengths and limitations of the meta-synthesis

To the best of our knowledge, this is the first review to explore qualitative research on the facilitators and barriers of lifestyle changes and lifestyle change maintenance among people with prediabetes. The application of a rigorous and systematic meta-synthesis technique with a transparent analytical procedure strengthens our paper. Synthesizing qualitative research is viewed as essential in achieving the goal of evidence-based practice and mainly features the use of the best available evidence as the foundation for this practice [75]. Another strength is that the included studies represent findings from several different countries with variously structured health systems. Despite this heterogeneity, we were able to identify many common themes, thus indicating how heterogeneity can be a strength rather than a limitation in a meta-synthesis [76].

A limitation to the meta-synthesis could be that the included articles were restricted to the English language, similar potential studies reported in other languages were consequently not retrieved nor appraised. Our included studies had no publication year limit, the oldest studies were conducted in 2008. However, in qualitative research one may argue that people’s experiences and perceptions on a specific topic are affected by context and the aspect of time to varying degrees. A meta-synthesis is a new and more comprehensive interpretation of already interpreted qualitative data from the primary studies [76], hence we did not use the raw data from the primary studies.

### Practical implications

The findings of these meta-synthesis might inform people with prediabetes, healthcare professionals and policy makers, in terms of the need for psychological, social, and environmental support for this population. More qualitative research is needed in this field to explore the reasons behind unhealthy behaviour and consider the complex interplay between all ecological levels influencing health behaviour. The translation of lifestyle intervention programs into practice seems to be limited since rates of type 2 diabetes are set to rise further. Considering this, it would be useful to pay more attention to the importance of the communication of risk and how people perceive risk and understand the diagnosis of prediabetes. This might provide insight into why people engage (or not) in lifestyle intervention programs for diabetes prevention. Lifestyle interventions in general seem to appeal more to those with greater resources and who can apply the appropriate information to improve health [77], therefore there is also a need for studies focusing on the effect of interventions for different groups in terms of socioeconomical status, culture, gender, and level of knowledge regarding prediabetes.

## Conclusion

This meta-synthesis offers important insights into evidence relevant to understanding the complexity and challenges of lifestyle change among people with prediabetes. Awareness of prediabetes and the perception of its related risks affects the motivation for lifestyle change; but this does not automatically lead to lifestyle changes. Facilitators and barriers for lifestyle change in people at risk for type 2 diabetes are found to be in a complex interplay within multiple levels of an ecological framework. Our findings illustrate how internal motivation and successful self-regulation facilitate lifestyle change and maintenance at the intrapersonal level. At the interpersonal level, social influence and support from family, peers, and health professionals comprise important facilitators; however, family and social norms can also represent barriers to change. Lifestyle intervention programs are important supportive contexts for lifestyle change, enhancing autonomy, competence and relatedness. Moreover, technological devices for monitoring lifestyle change could provide support for those not participating in a lifestyle intervention programme. The environmental and policy levels set the foundations for the availability of health promoting options and plays a crucial role in shaping the conditions for successful lifestyle change. A purely individual approach is far from sufficient in combating the rising global epidemic of type 2 diabetes. A great responsibility lies on health authorities and policymakers to create health-promoting environments.

## Supplementary Information

**Additional file 1. **Example of search strategy.

## Data Availability

All data generated or analysed during this study are included in this published article. The data presented in our review are retrieved from the published papers of the included studies.

## Abbreviations

IFG: Impaired fasting glucose
IGT: Impaired glucose tolerance
FPG: Fasting plasma glucose
FBG: Fasting blood glucose
OGTT: Oral glucose tolerance test
BMI: Body mass index
T2D: Type 2 diabetes
DPP: Diabetes prevention program
