# Supplementary material for: Facilitators and barriers for lifestyle change in people with prediabetes: a meta-synthesis of qualitative studies
Source: BMC Public Health. 2022 Mar 21;22:553. doi: 10.1186/s12889-022-12885-8 (PMC8935766; doi:10.1186/s12889-022-12885-8)
Supplement: Supplementary file 1 — Additional file 1. Example of search strategy. [file 12889_2022_12885_MOESM1_ESM.docx]

**Additional File 1**

Example of search strategy in Medline (27 April 2021)

1. adult/ or aged/ or "aged, 80 and over"/ or middle aged/ or young adult/

2. (adult* or aged or elderly or senior*).tw,kf,kw.

3. Prediabetic State/

4. (prediabetic or prediabetes or pre-diabetes).tw,kw,kf.

5. hyperglycemia/ or glucose intolerance/ or hyperinsulinism/ or insulin resistance/ or metabolic syndrome/

6. (hyperglyc* or glucose intolerance or hyperinsulinism or insulin resistance or metabolic syndrome or fasting glucose or impair* glucose tolerance or impair* fasting glycemia).tw,kw,kf.

7. Risk Factors/ and Diabetes Mellitus,Type 2/

8. 3 or 4 or 5 or 6 or 7

9. 1 or 2

10. 8 and 9

11. health behavior/ or health risk behaviors/ or risk reduction behavior/ or sedentary behavior/ or life style/ or healthy lifestyle/ or life change events/

12. (health behavi* or health risk behavi* or risk reduction behavi* or sedentary behavi* or life style or lifestyle or healthy life style or healthy lifestyle or life change event*).tw,kw,kf.

13. health education/ or healthy people programs/ or weight reduction programs/ or patient education as topic/ or motivational interviewing/

14. (health education or healthy people program* or weight reduction program* or motivational interviewing or patient education or (physical education adj1 training) or health knowledge or (attitudes adj1 health)).tw,kf,kw.

15. ((health behavior* or life style* or lifestyle*) adj3 (intervention* or program* or change* or modificat* or counsel*)).tw,kw,kf.

16. peer group/ or peer influence/ or group process/ or interpersonal relation/ or nurse-patient relation/ or physician-patient relation/ or therapeutic alliance/

17. (peer group* or peer influence* or group process* or interpersonal relation* or nurse-patient relation* or physician-patient relation* or therapeutic alliance).tw,kf,kw.

18. Self efficacy/ or empowerment/ or motivation/ or goal/ or mental health/ or awareness/ or comprehension/ or consciousness/ or perception/ or personal autonomy/

19. (self efficacy or self-efficacy or empowerment or motivation* or goal* or mental health* or awareness or comprehension or consciousness or perception* or personal autonomy).tw,kf,kw.

20. exp diet/ or diet therapy/

21. (diet* or healthy eating or healthy nutrition).tw,kf,kw.

22. exp exercise/ or exercise therapy/

23. (exercis* or physical activit* or training* or active living or walking or outdoor activit*).tw,kf,kw.

24. 11 or 12 or 13 or 14 or 15 or 16 or 17 or 18 or 19 or 20 or 21 or 22 or 23

25. Qualitative Research/ or Focus Groups/ or Grounded Theory/ or Interviews as Topic/ or Narration/ or Personal Narrative/ or Interview/

26. (qualitative research or qualitative stud* or focus group* or interview* or semi-structured interview* or unstructured interview* or narration or narrative or hermeneutic* or phenomenolog* or phenomenological research or ethnographic research or thematic analys* or content analys* or grounded theory or experience* or facilitator* or barrier*).tw,kw,kf.

27. 25 or 26

28. 10 and 24 and 27

29. 11 or 12 or 15 or 20 or 21 or 22 or 23

30. 10 and 27 and 29
